# IL-35 induces N2 phenotype of neutrophils to promote tumor growth

**DOI:** 10.18632/oncotarget.16819

**Published:** 2017-04-04

**Authors:** Jiu-Ming Zou, Jian Qin, Yong-Chao Li, Yu Wang, Dong Li, Yu Shu, Chao Luo, Shan-Shan Wang, Gang Chi, Fang Guo, Gui-Mei Zhang, Zuo-Hua Feng

**Affiliations:** ^1^ Department of Biochemistry and Molecular Biology, School of Basic Medicine, Tongji Medical College, Huazhong University of Science and Technology, Wuhan 430030, The People's Republic of China

**Keywords:** IL-35, neutrophils, inflammation, tumor, cytokine

## Abstract

IL-35 is an immunosuppressive cytokine and exerts regulatory effects on T cells, B cells, macrophages and dendritic cells. Neutrophils are important innate immune cells that play key roles in tumor development. The effect of IL-35 on neutrophils remains unknown. Here, we report that IL-35 can induce N2 neutrophil polarization (protumor phenotype) by increasing G-CSF and IL-6 production, and promote neutrophil infiltration into tumor microenvironment. The sustained expression of IL-35 could promote chronic inflammation to augment the proangiogenic and immunosuppressive function of neutrophils. IL-35 stimulated macrophages to secrete proinflammatory cytokines IL-1β and IL-6. IL-1β stimulated γδ T cells to produce IL-17, which in turn increased the production of G-CSF. By increasing the expression of G-CSF and IL-6, IL-35 could up-regulate the expression of MMP-9 and Bv8, and down-regulate TRAIL expression in neutrophils, thus augmenting the proangiogenic function of neutrophils. Moreover, G-CSF/IL-6 induced the enhanced activation of STAT3 and ERK pathways in neutrophils, thus increasing the expression of iNOS to suppress T cell activation. Our findings suggest that IL-35 can promote tumor progression by functioning as an up-stream cytokine to promote cancer-associated inflammation and control neutrophil polarization. Targeting IL-35 might be an important approach for designing new strategy of tumor therapy.

## INTRODUCTION

Inflammation, especially chronic inflammation, is a driving force for tumor initiation and progression. Tumor cells themselves often express some inflammatory cytokines. These inflammatory cytokines play important roles in the processes of cancer-related inflammation [1]. IL-35 is a recently discovered heterodimeric cytokine composed of p35 and EBI3 subunits [2]. Although IL-35 was found as an anti-inflammatory cytokine that inhibits the differentiation of Th17 cells in murine models of autoimmune diseases [3, 4], other studies showed the pro-inflammatory effects of IL-35 in murine models of collagen-induced arthritis and Lyme arthritis [5, 6]. Importantly, IL-35 has been found to promote tumor progression and metastasis [7, 8]. Clinical evidences showed that the higher expression of p35 and EBI3 was detected in tumor tissues [9]. The elevated serum IL-35 levels correlated with poor prognosis in patients with renal cell carcinoma and non-small cell lung cancer [10, 11]. Given that a chronic inflammatory microenvironment is an essential component of all of tumors [1], it is possible that IL-35 may promote tumor development by facilitating chronic inflammation.

Neutrophils are the important components of inflammatory response, which have dual roles in tumor development and metastasis. In response to the stimulation of different cytokines, neutrophils have the potential to polarize towards antitumor (N1) or protumor (N2) phenotypes [12]. In the acute inflammation state, neutrophils are activated to exert antitumor effect. On the contrary, neutrophils are activated by chronic inflammation to promote tumor growth and metastasis. The inflammatory cytokines such as G-CSF, IL-6 and TGF-β1 can induce N2 phenotype of neutrophils in bone marrow and tumor microenvironment [13, 14]. In addition, priming with IFN-γ and TNF-α can convert the phenotype of neutrophils from N2 to N1 [15]. Thereby, neutrophils may exert either antitumor or protumor function, which mainly depends on regulation of inflammatory cytokines.

A recent report has shown that IL-35 promoted tumor growth and metastasis in Rag1/2-deficient mice that lack T and B lymphocytes [16], suggesting that IL-35 may also regulate the innate immune system to promote tumor development. Neutrophils are the most common component of the innate immune system. IL-35 has been demonstrated to possess an immunoregulatory potential on T and B lymphocytes [17, 18], but the effects of IL-35 on neutrophils remains unknown. In the present study, we investigated whether IL-35 might modulate the polarization of neutrophils to promote tumor progression. Our data showed that IL-35 could function as an up-stream cytokine to promote cancer-associated inflammation, and that IL-35 could induce the polarization of neutrophils towards N2 phenotype, thus facilitating tumor development.

## RESULTS

### IL-35 promotes neutrophil infiltration in tumor microenvironment

**Figure 1:**
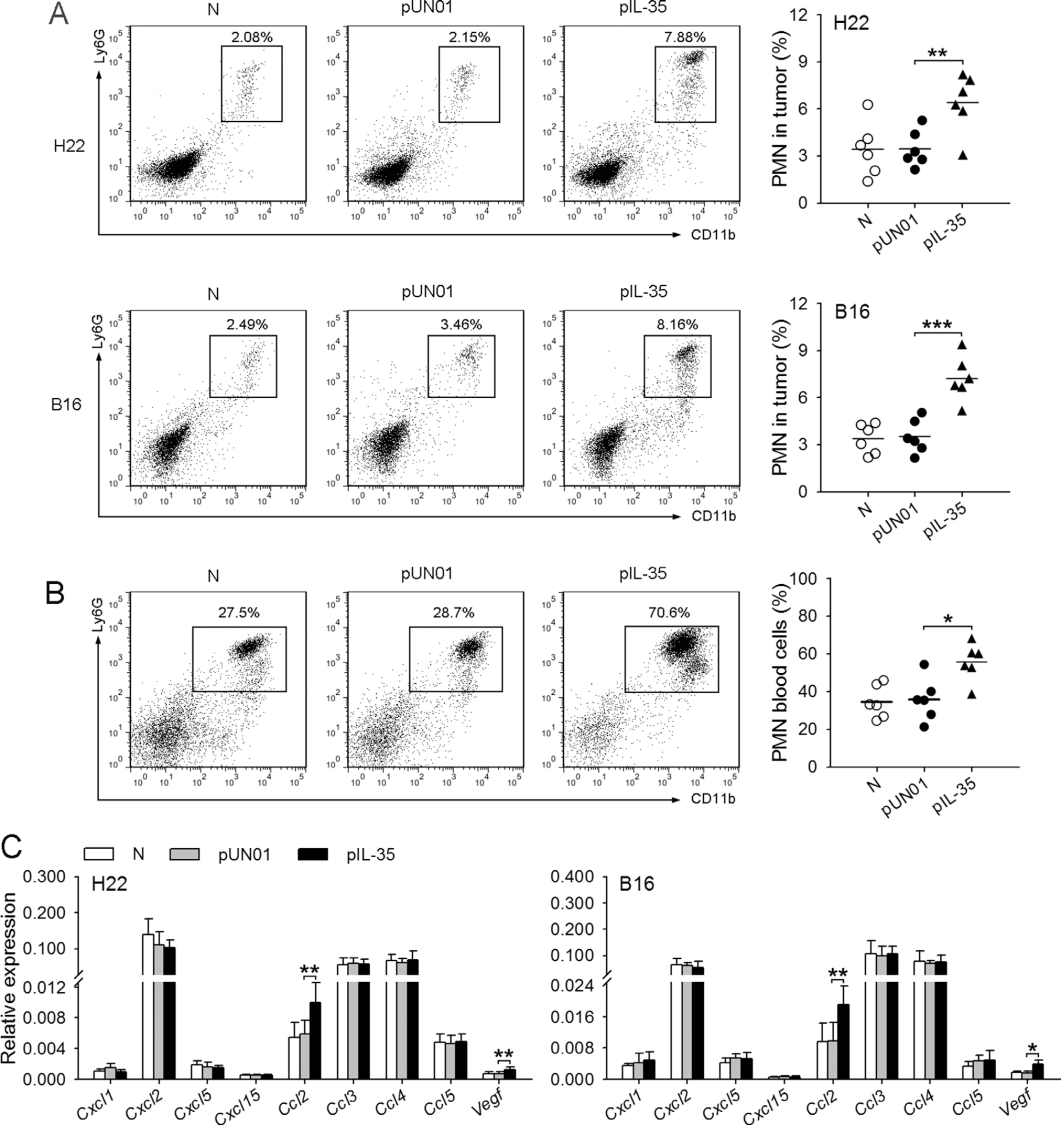
IL-35 promotes neutrophil infiltration in tumor microenvironment (**A**) Tumor cells were intramuscularly injected to the right hind thigh of naive mice (N), pUN01-mice and pIL-35-mice. The plasmid injection was continued after tumor cell inoculation, once every two days. Tumors were dissected on day 10 after tumor cell inoculation. Neutrophils in tumor tissues were analyzed by flow cytometry. (**B**) Flow cytometric analysis of neutrophils in peripheral blood of naive mice, pUN01-mice, and pIL-35-mice. (**C**) In the experiments same as (A), the expression of *Cxcl1*, *Cxcl2*, *Cxcl5*, *Cxcl15*, *Ccl2*, *Ccl3*, *Ccl4*, *Ccl5*, and *Vegf* genes in tumor tissues was detected at the mRNA level by real-time RT-PCR. Data are pooled from three independent experiments with a total six samples in each group. *p < 0.05, **p < 0.01, ***p < 0.001.

To explore the effect of IL-35 on neutrophils *in vivo*, we fist investigated whether IL-35, as part of its effect on inflammation, may influence neutrophil infiltration in tumor microenvironment. For this purpose, we analyzed the amount of neutrophils in the tumor tissues after the inoculation of tumor cells to naive mice and the mice with *in vivo* transfection of IL-35 expression vector (IL35-mice). The results showed that the amount of neutrophils in the tumor tissues from IL35-mice was significantly higher than that in the tumor tissues from naive mice (Figure 1A). Consistently, the proportion of neutrophils in blood cells in IL35-mice was significantly increased, compared with that in control mice (Figure 1B), indicating that the *in vivo* expression of IL-35 promoted the mobilization of neutrophils. Moreover, we also detected the gene expressions of chemoattractants for neutrophils in tumor microenvironment, including CXCL1, CXCL2, CXCL5, CXCL15, CCL2, CCL3, CCL4, CCL5 and VEGF [19–21]. The results showed that several genes were highly expressed in tumor, including *Cxcl2*, *Ccl2*, *Ccl3*, *Ccl4*, and *Ccl5* (Figure 1C). These results suggested that IL-35 might promote the infiltration of neutrophils by increasing circulating neutrophils, based on the expression of multiple chemoattractants for neutrophils in tumor microenvironment.

To further ascertain the effect of IL-35, we inoculated the mice with B16F0 cells expressing IL-35 (B16-IL35), or inoculated the mice with H22 cells followed by local transfection of pIL-35 to express IL-35 in tumor microenvironment. Consistent with the above results, the increase of local IL-35 expression in tumor promoted the growth of tumor and the infiltration of neutrophils (Supplementary Figure 1A and 1B).

### IL-35 switches the function of neutrophils toward tumor-promoting

**Figure 2:**
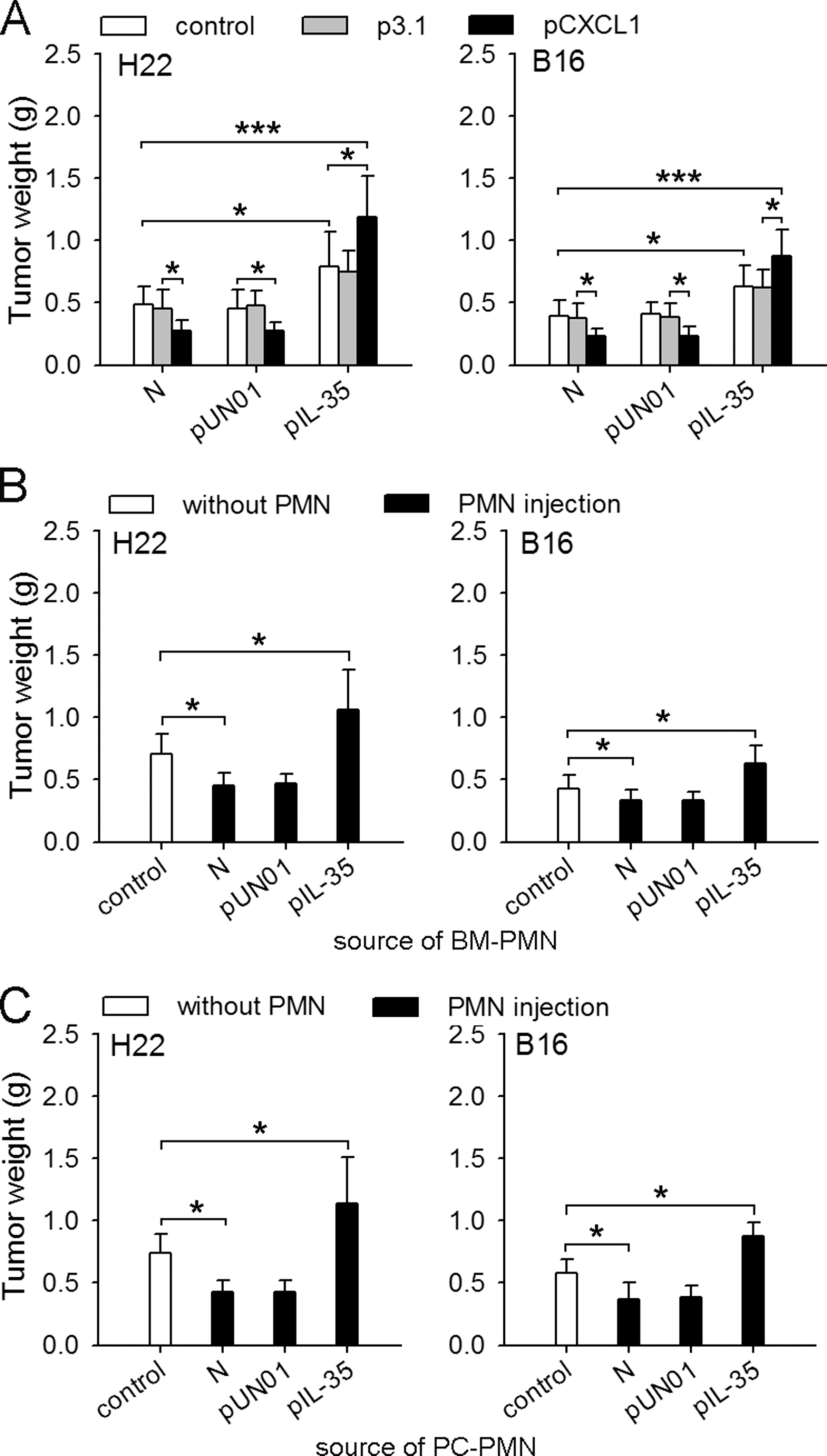
IL-35 switches the function of neutrophils toward tumor-promoting (**A**) H22 or B16 cells were inoculated into naive mice, pUN01-mice, and pIL-35-mice. CXCL1 was expressed in local tissues to recruit neutrophils, as described in Methods. The effect of neutrophil recruitment on tumor growth was evaluated by measuring tumor weight. (**B** and **C**) Neutrophils were isolated from bone marrow (BM) or peritoneal cavity (PC) of naive mice, or pUN01-mice and pIL-35-mice. The neutrophils from BM (B) or PC (C) were used for co-inoculation test as described in Methods. Tumors (*n* = 9 per group) were dissected and weighed on d10 after tumor cell inoculation. *p < 0.05, **p < 0.01, ***p < 0.001.

We next investigated the effect of IL-35 on the function of neutrophils *in vivo* by recruiting neutrophils to the tissues at the inoculation site in naive mice and IL35-mice. The recruitment of neutrophils suppressed the growth of tumor in naive mice, but significantly promoted tumor growth in IL35-mice (Figure 2A), suggesting that IL-35 could induce the conversion of neutrophil function *in vivo*. To further confirm this, we isolated neutrophils from bone marrow of mice to test their function. In co-inoculation test, the neutrophils from IL35-mice indeed significantly promoted the growth of tumor (Figure 2B). To ascertain that the protumor function of neutrophils could be maintained after the process of chemotaxis, we isolated neutrophils from peritoneal cavity of mice after recruitment, and tested their function in co-inoculation test. The results showed that the neutrophils from naive mice suppressed the growth of tumor, but the neutrophils from IL35-mice promoted tumor growth (Figure 2C). Taken together, these results indicated that the sustained expression of IL-35 *in vivo* could induce the conversion of neutrophil function from tumor-suppressing to tumor-promoting.

### IL-35 augments proangiogenic and immunosuppressive function of neutrophils *in vivo*

**Figure 3:**
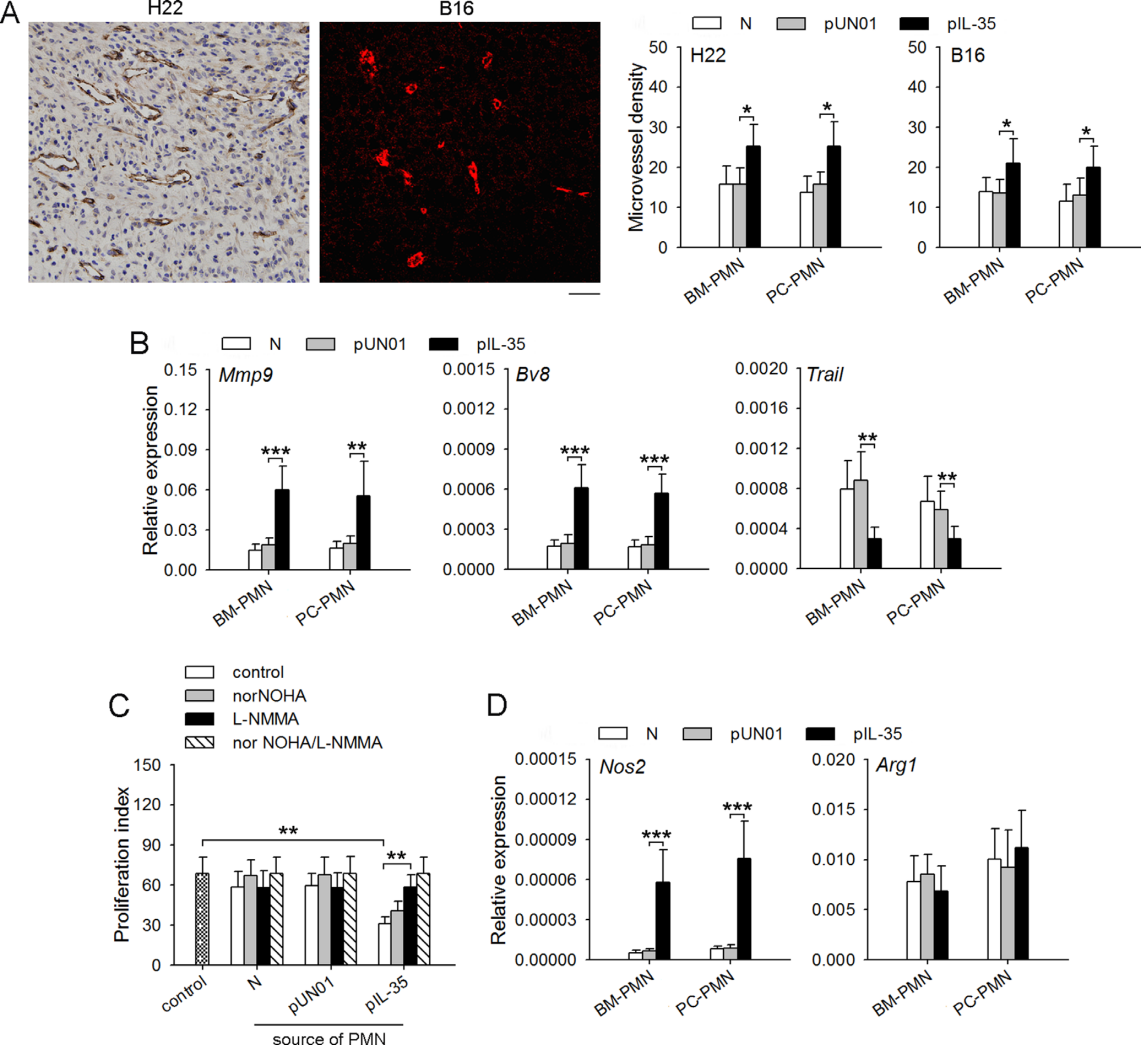
IL-35 augments proangiogenic and immunosuppressive function of neutrophils *in vivo* (**A**) Neutrophils were isolated from bone marrow (BM) or peritoneal cavity (PC) of naive mice, pUN01-mice, and pIL-35-mice. The neutrophils were injected into palpable tumors. Microvessels in tumor tissues were detected by immunohistochemical analysis (left, scale bar, 50 um). Microvessel density was determined as described in Methods (right). (**B**) Neutrophils were isolated from bone marrow or peritoneal cavity of naive mice, pUN01-mice, and pIL-35-mice. The expression of *Mmp-9*, *Bv8*, and *Trail* genes in BM and PC neutrophils were detected by real-time RT-PCR. (**C**) CFSE-labeled splenocytes from naive mice were incubated with CD3/CD28 stimulation beads in the absence or presence of the neutrophils isolated from bone marrow of naive mice, pUN01-mice and pIL-35-mice. NorNOHA and L-NMMA were added as indicated. After 48-h culture, splenocyte proliferation was measured as described in Methods. (**D**) Neutrophils were isolated from bone marrow or peritoneal cavity of naive mice, pUN01-mice, and pIL-35-mice. The expression of *Nos2* and *Arg1* genes in neutrophils were detected by real-time RT-PCR. Data are pooled from three independent experiments with a total six samples in each group. *p < 0.05, **p < 0.01, ***p < 0.001.

Neutrophils with protumor phenotype could promote tumor development by promoting tumor angiogenesis and suppressing immune response [22, 23]. To further ascertain the conversion of neutrophil function, we analyzed the effect of IL-35 on the proangiogenic and immunosuppressive function of neutrophils. The results showed that neutrophils from IL35-mice could significantly promote tumor angiogenesis (Figure 3A). Similar results were obtained when IL-35 was expressed in tumor microenvironment (Supplementary Figure 1C). Consistently, the sustained expression of IL-35 *in vivo* resulted in up-regulation of expression levels of MMP-9 and Bv8 that promote angiogenesis, and the down-regulation of TRAIL that suppresses angiogenesis in neutrophils (Figure 3B and Supplementary Figure 2A, 2B).

We then analyzed whether IL-35 might augment the immune suppressive function of neutrophils. When splenic cells were stimulated with CD3/CD28 antibodies in the presence of neutrophils, T cell proliferation was not significantly suppressed by the neutrophils from naive mice. However, the neutrophils from IL35-mice significantly suppressed T cell proliferation (Figure 3C and Supplementary Figure 2C), indicating that the sustained expression of IL-35 significantly augmented the immunosuppressive function of neutrophils. Neutrophils have been known to produce arginase 1 (Arg-1) and inducible nitric oxide synthase (iNOS) to suppress T cell antitumor immune response [24–26]. Arg-1 was involved in the inhibitory effect of neutrophils from naive mice, since the effect could be abolished by arginase-1 inhibitor, norNOHA (Figure 3C). Intriguingly, IL-35-augmented suppressive function of neutrophils was abrogated by iNOS inhibitor, L-NMMA (Figure 3C), suggesting that IL-35 could augment the immunosuppressive function of neutrophils by increasing the expression of iNOS. To further confirm this, we analyzed the expression of *Arg1* and *Nos2* genes. In the neutrophils from naive mice, the expression of Arg-1, but not iNOS, was detectable (Supplementary Figure 2D). In the neutrophils from IL35-mice, the expression of iNOS, but not Arg-1, was significantly increased (Figure 3D and Supplementary Figure 2D), suggesting that IL-35 could augment the immunosuppressive function of neutrophils by increasing iNOS expression.

### G-CSF/IL-6 but not IL-35 directly alters the function of neutrophils

**Figure 4:**
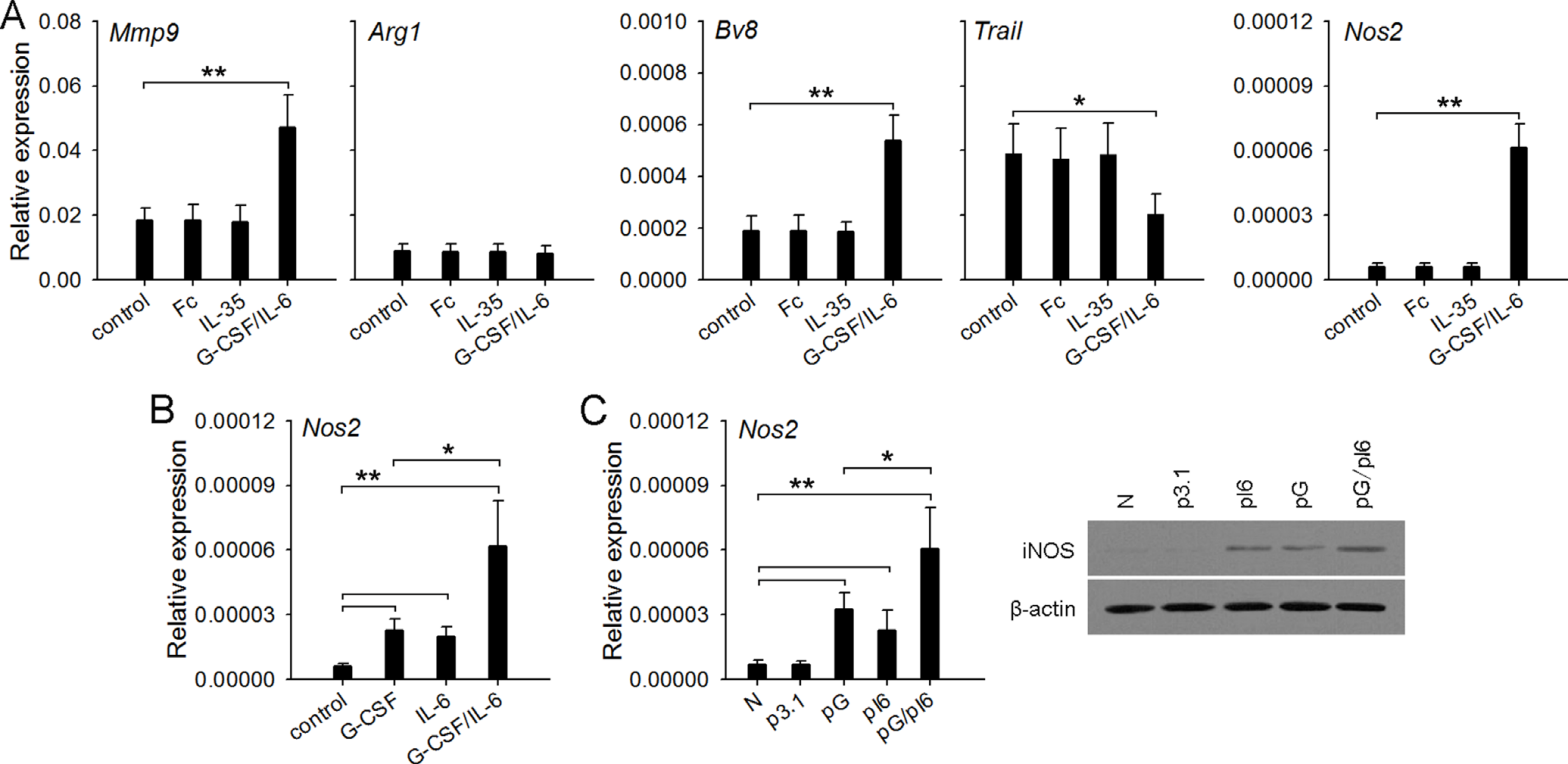
G-CSF/IL-6 but not IL-35 directly alters the function of neutrophils (**A**) Neutrophils were isolated from naive mice and cultured (2 × 10^6^/ml) in 6-well flat bottom plate (2 ml/well) in the absence or presence of IL-35 (100 ng/ml, or G-CSF/IL-6 (50 ng/ml of each). After 12-h culture, gene expressions were detected by real-time PCR. The IgG Fc fragment (Fc) was using as control for excluding any nonspecific effect of recombinant protein. (**B**) Neutrophils were isolated from bone marrow of naive mice, and cultured in the absence or presence of G-CSF and/or IL-6 (50 ng/ml of each). After 12-h culture, the expression of Nos2 gene was detected. (**C**) Neutrophils were isolated from BM of naive mice, p3.1-mice, pG-mice, pI6-mice, and pG/pI6-mice. The expression of *Nos2* gene in neutrophils was detected by real-time RT-PCR and western blots. Data are representative of three independent experiments (C, right), or are pooled from three independent experiments with a total six samples in each group (A–C). *p < 0.05, **p < 0.01, ***p < 0.001.

Next, we wondered whether IL-35 could directly induce the conversion of neutrophil function. To clarify this, we isolated neutrophils from naive mice, and stimulated the cells with recombinant mouse IL-35. Unexpectedly, the stimulation with IL-35 did not influence the expression of *Mmp9*, *Bv8*, *Trail*, *Nos2*, and *Arg1* genes in neutrophils (Figure 4A), suggesting that IL-35 could not directly induce the conversion of neutrophil function. Our previous study showed that G-CSF and IL-6 could up-regulate the expression of *Mmp9* and *Bv8*, and down-regulate the expression of *Trail* [13]. Interestingly, G-CSF/IL-6 not only modulated the expression of these genes, but also significantly increased the expression of *Nos2* gene (Figure 4A). Both G-CSF and IL-6 alone could induce the expression of *Nos2* gene, and cooperatively induce higher expression of *Nos2* by directly stimulating neutrophils (Figure 4B). Likewise, *in vivo* transfection of G-CSF and IL-6 expression vectors enhanced the expression level of *Nos2* gene in bone marrow neutrophils (Figure 4C).

### Enhanced activation of STAT3 and ERK pathways is required for upregulating iNOS

**Figure 5:**
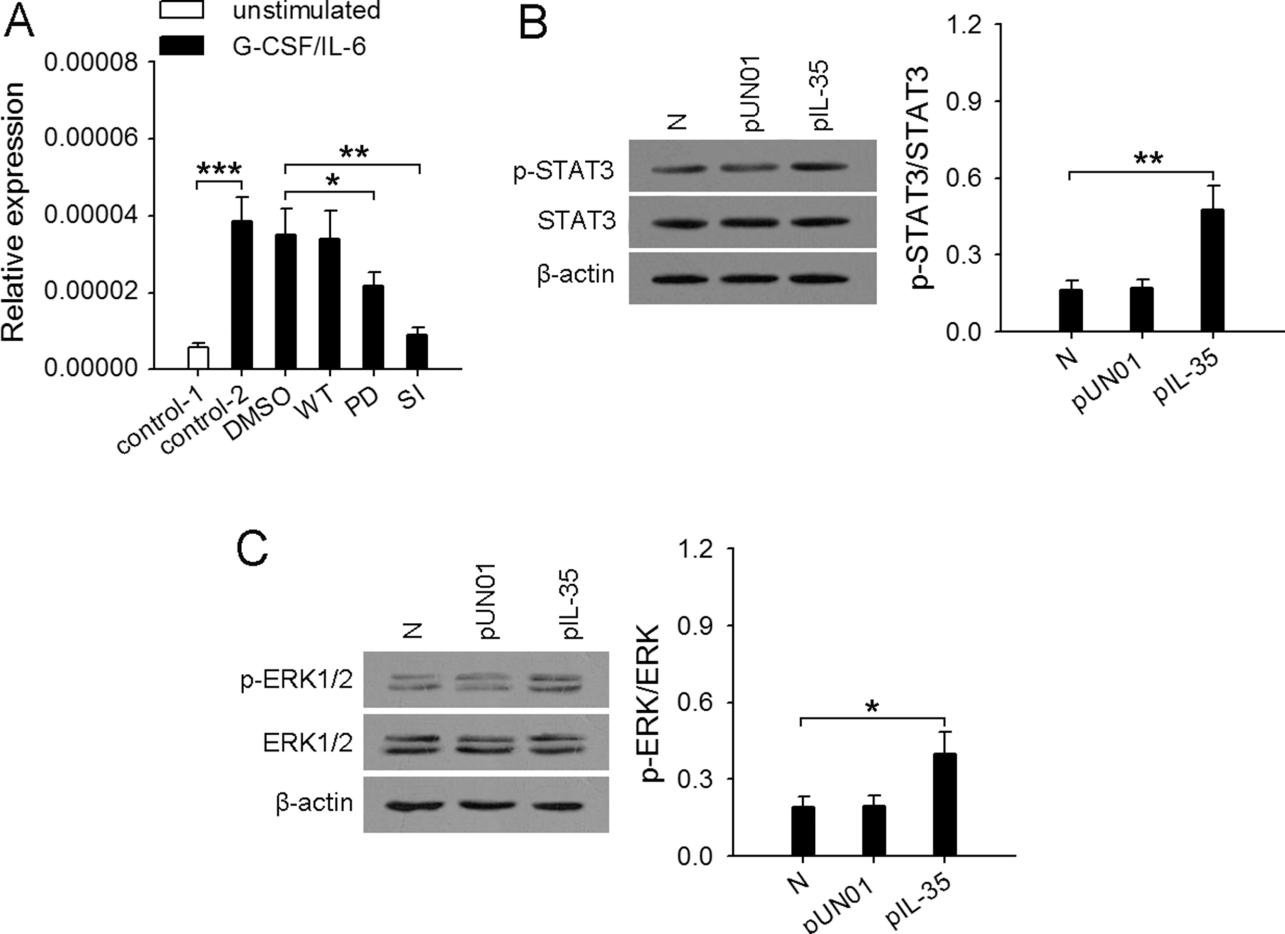
Enhanced activation of STAT3 and ERK pathways in neutrophils is required for the upregulation of iNOS (**A**) Neutrophils were isolated from bone marrow of naive mice. The cells (2 × 10^6^/ml) were untreated or pretreated for 2 h with STAT3 inhibitor VIII (SI, 50 μM), wortmannin (WT, 10 nM), PD98059 (PD, 20 μM), and then stimulated for 12 h with or without G-CSF/IL-6 (50 ng/ml of each) in the absence or presence of the same inhibitor. The expression of *Nos2* gene was detected at the mRNA level by real-time RT-PCR. (**B** and **C**) Neutrophils were isolated from bone marrow of naive mice, pUN01-mice, and pIL-35-mice. The phospho-STAT3 (p-STAT3) and total STAT3 (B), phospho-Erk1/2 (p-Erk1/2) and total Erk1/2 (C) in neutrophils were detected by Western blot. The ratios of (p-STAT3/STAT3) or (p-ERK/ERK) were calculated after densitometric analysis of Western blots. Data are representative of three independent experiments (B, left; C, left), or pooled from three independent experiments with a total six samples in each group. *p < 0.05, **p < 0.01.

Our previous study showed that G-CSF/IL-6 could modulate the expression of *Mmp9*, *Bv8*, and *Trail* by inducing the enhanced activation of STAT3 pathways [13]. Here we further investigated the mechanism underlying the up-regulation of *Nos2* gene. It has been reported that the expression of iNOS in neutrophils could be regulated by several signaling pathways, including p38 MAPK, JNK, ERK [27, 28]. However, G-CSF mainly activates the STAT3, PI3K, and ERK pathways in neutrophils [29, 30]. IL-6 also activates STAT3 pathway [31]. We therefore stimulated neutrophils with G-CSF and/or IL-6 in the presence of wortmannin (PI3K inhibitor), PD98059 (inhibitor of ERK pathway), or STAT3 inhibitor VIII. The results showed that *Nos2* gene expression was suppressed in the presence of STAT3 inhibitor VIII, when neutrophils were stimulated by G-CSF and/or IL-6 (Figure 5A and Supplementary Figure 3). Inhibiting ERK pathway with PD98059 could partially inhibit *Nos2* gene expression in the presence of G-CSF, but inhibiting PI3K pathway with wortmannin did not influence the expression of *Nos2* gene. These results indicated that STAT3 and ERK pathways were involved in G-CSF/IL-6-induced expression of *Nos2* gene. Consistently, the phosphorylation levels of STAT3 and ERK1/2 in the neutrophils from IL35-mice were significantly higher than those in the neutrophils from naive mice (Figure 5B, 5C).

### IL-35 increases G-CSF and IL-6 production *in vivo*

**Figure 6:**
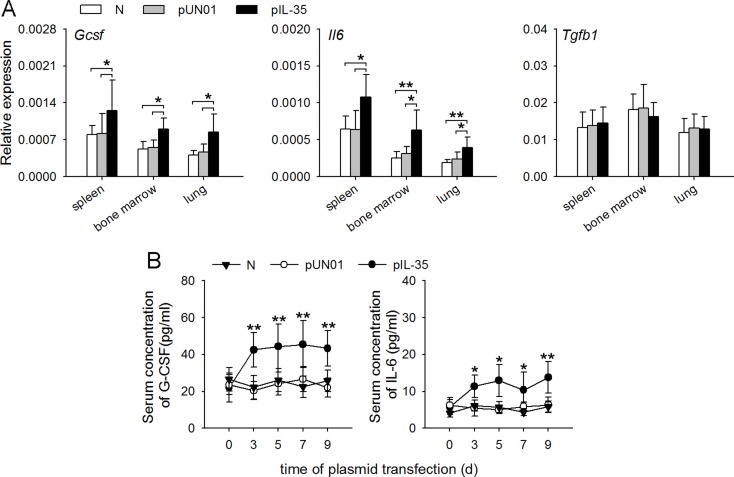
IL-35 increases G-CSF and IL-6 production *in vivo* (**A**) The expressions of *Gcsf*, *Il6* and *Tgfb1* genes in the indicated organs of naive mice, pUN01-mice, and pIL-35-mice were detected by real-time RT-PCR. (**B**) Mice were untreated or received i.m. injection of pIL-35 plasmid or pUN01 plasmid. Then, the G-CSF and IL-6 levels in serum of mice were detected by ELISA at the indicated time point. Data are pooled from three independent experiments with a total six samples in each group. * *p* < 0.05, ** *p* < 0.01.

The above results suggested that IL-35 might modulate gene expression in neutrophils by inducing the expression of G-CSF and IL-6. To ascertain this, we investigated whether IL-35 might influence the *in vivo* expression of G-CSF, IL-6 and TGF-β that are able to regulate the function of neutrophils. After the sustained expression of IL-35, the expression levels of *Gcsf* and *Il6* mRNAs, but not *Tgfb1* mRNA, were significantly increased in the spleen, lung and bone marrow of mice (Figure 6A). Consistently, IL-35 induced the persistently low and steady levels of G-CSF and IL-6 in blood (Figure 6B).

### IL-35 stimulates macrophages to produce IL-6 but not G-CSF

**Figure 7:**
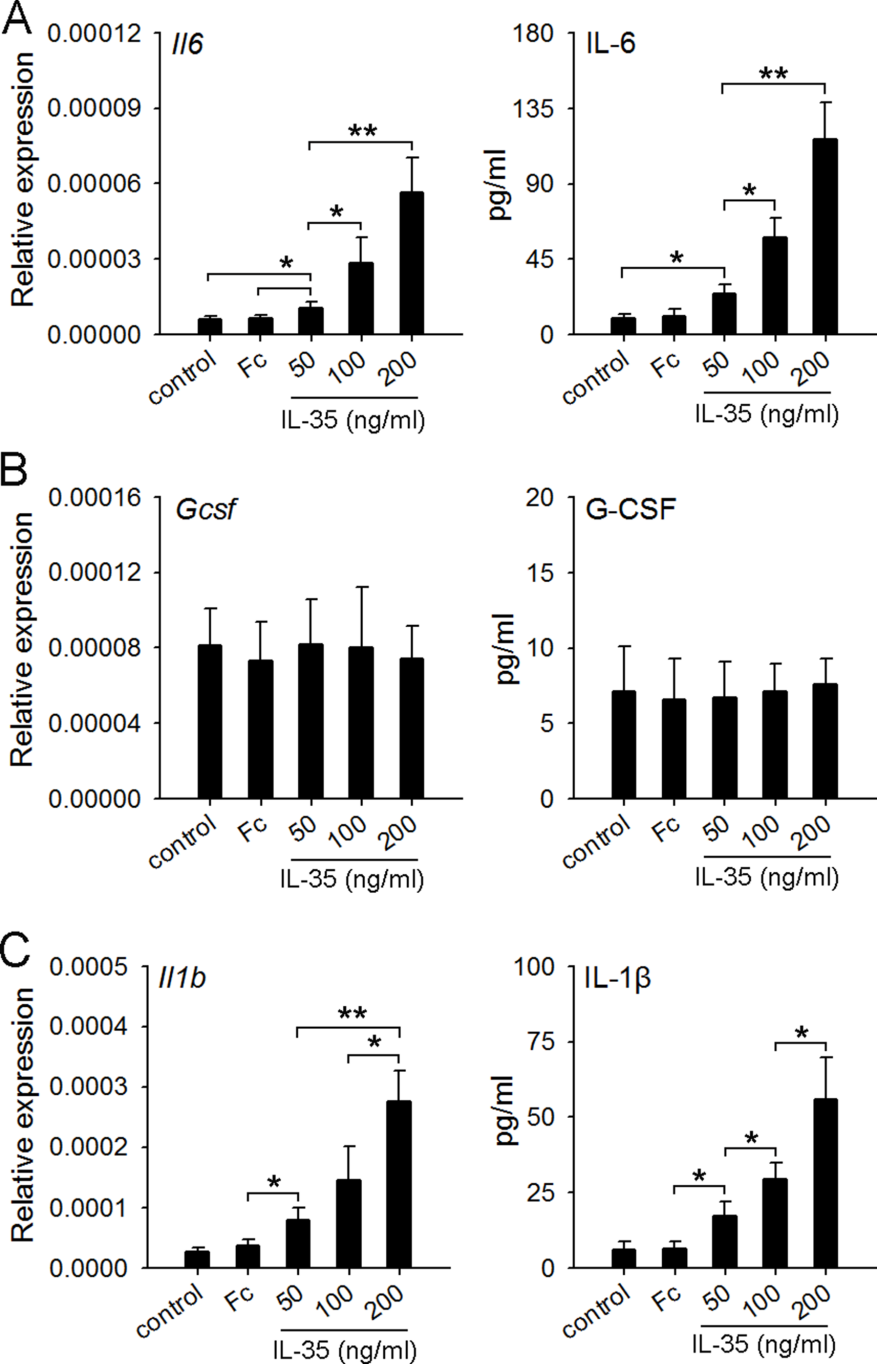
IL-35 stimulates macrophages to produce IL-6 but not G-CSF (**A**–**C**) Macrophages were prepared from naive mice as described in Methods. The cells were then cultured in the absence or presence of IL-35 at the indicated concentration. IgG Fc fragment (Fc) was used as control. After 12-h culture, the expressions of *Il6* (A) *Gcsf* (B) and *Il1b* (C) genes were detected by real-time PCR. The levels of IL-6, G-CSF and IL-1β in culture supernatants were detected by ELISA. Data are pooled from three independent experiments with a total six samples in each group. *p < 0.05, **p < 0.01.

A recent study showed that recombinant human IL-35 cytokine could stimulate peripheral blood mononuclear cells (PBMCs) to produce IL-6 and IL-1β [32]. IL-6 could be produced by macrophages and the activated T lymphocytes [33]. Macrophages were also an important source of IL-1β and G-CSF [24, 34]. Thus, we prepared macrophages from the naive mice, and detected the expression of IL-6, IL-1β and G-CSF after the stimulation with IL-35. The results showed that IL-35 dose-dependently induced the expression of IL-6 and IL-1β, but not G-CSF (Figure 7), suggesting that IL-35 could directly stimulate macrophages to produce IL-6, but induce the G-CSF production *in vivo* in a different way.

### Macrophages/IL-1β/γδ T cells/IL-17 axis is involved in IL-35-induced expression of G-CSF *in vivo*

**Figure 8:**
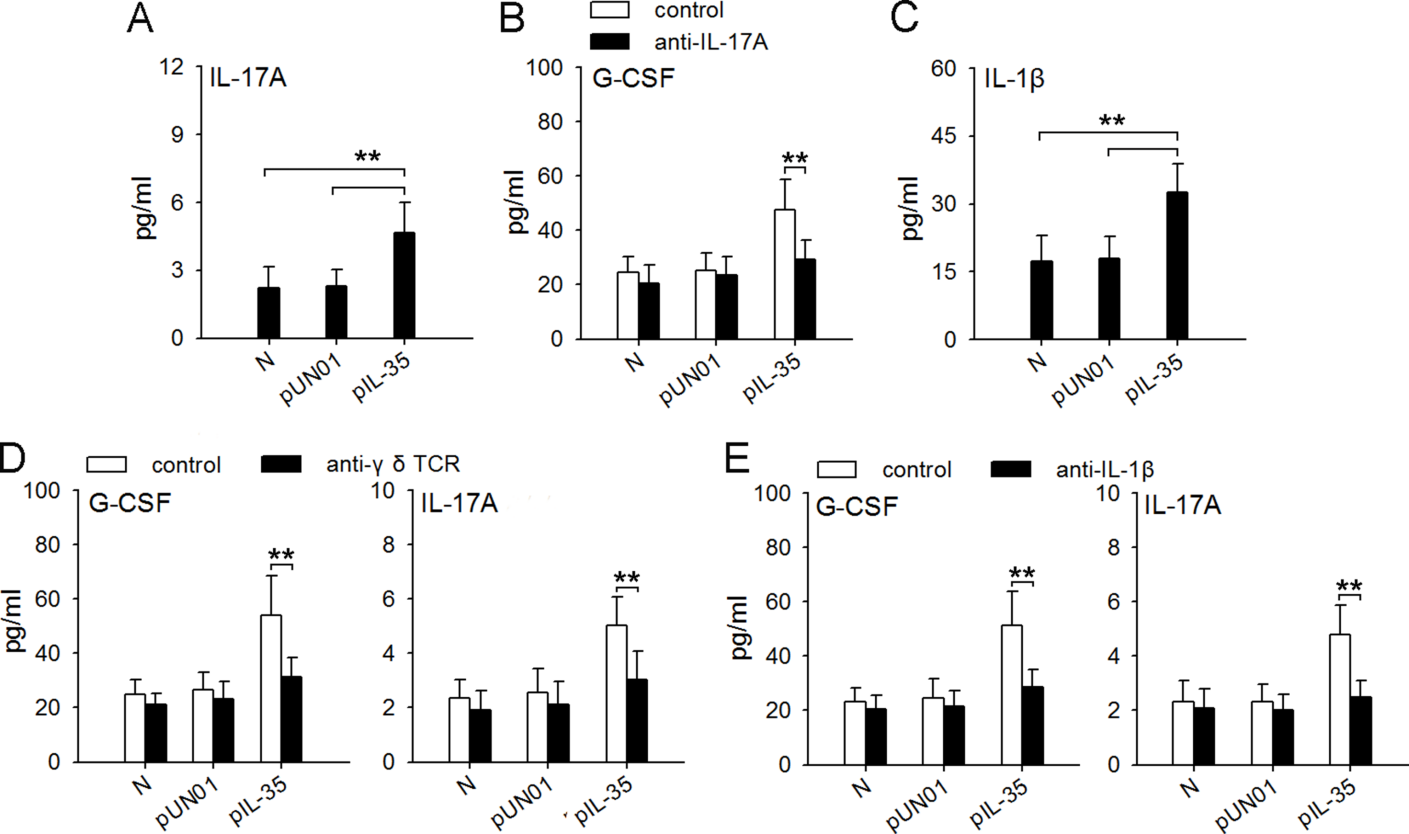
Macrophages/IL-1β/γδT cells/IL-17 axis is involved in IL-35-induced expression of G-CSF *in vivo* (**A**) ELISA analysis of IL-17A in the serum of naive mice, pUN01-mice and pIL-35-mice. (**B**) Mice were untreated or treated with anti-IL-17A antibody to neutralize IL-17A *in vivo*. G-CSF levels in serum of naive mice, pUN01-mice, and pIL-35-mice were detected by ELISA. (**C**) ELISA analysis of IL-1β in the serum of naive mice, pUN01-mice and pIL-35-mice. (**D**) After depletion of γδ T cells with anti-γδ TCR antibody *in vivo*, the levels of G-CSF and IL-17A in serum of naive mice, pUN01-mice, and pIL-35-mice were detected by ELISA. (**E**) Mice were untreated or treated with anti-IL-1β antibody to neutralize IL-1β *in vivo*. G-CSF and IL-17A levels in serum of naive mice, pUN01-mice, and pIL-35-mice were detected by ELISA. **p < 0.01.

It has been known that IL-17A could induce the production of G-CSF *in vivo* [24]. Interestingly, IL-17A level in the blood of IL35-mice was much higher than that in the blood of naive mice (Figure 8A). In addition to IL-6, the increase of IL-35 expression in tumor could increase IL-17A and G-CSF in blood and tumor microenvironment (Supplementary Figure 4). We therefore investigated whether IL-35 might induce G-CSF expression *in vivo* by increasing IL-17A expression by neutralizing IL-17A *in vivo*. Indeed, the neutralization of IL-17A *in vivo* suppressed the production of G-CSF in IL35-mice (Figure 8B). CD4^+^Th17 cells and γδ T cells are two populations that produce IL-17A [35]. Recent studies showed that IL-1β/γδ T cells axis was important regulatory pathway of IL-17A [24]. In IL35-mice, the frequency of IL-17A-producing CD4^+^Th17 cells in spleen was not significantly changed (Supplementary Figure 5), but the serum level of IL-1β was significantly increased (Figure 8C), suggesting that γδ T cells might be the source of IL-17A in presence of IL-35. In line with this, depleting γδ T cells (Figure 8D) or neutralizing IL-1β (Figure 8E) *in vivo* could abrogate the increased expression of both G-CSF and IL-17A in IL35-mice. Taken together, these results suggest that macrophages/IL-1β/γδ T cells/IL-17 axis is involved in IL-35-induced expression of G-CSF *in vivo*.

## DISCUSSION

High expression of IL-35 in tumor tissues and the elevated plasma IL-35 levels have been reported in patients with solid or hematopoietic malignancy [10, 11, 36]. The increase of IL-35 in tumor could promote tumor progression as shown by our data and others [37]. Moreover, our data also showed that the increase of IL-35 in blood resulted in N2 polarization of neutrophils, thus promoting tumor progression. IL-35 could function as an up-stream cytokine to control neutrophil polarization by inducing the production of other cytokines. Although IL-35 might not be the only cytokine that induces the conversion of neutrophil function from tumor-suppressing to tumor-promoting, the increase of IL-35 in tumor and blood could certainly promote the tumor-promoting function of neutrophils.

Neutrophils, as an important population of inflammatory cells, may have different phenotypes in different inflammatory disorders. Acute inflammation induces N1 neutrophils to eliminate pathogens, whereas chronic inflammation induces N2 neutrophils to promote angiogenesis and tissue remodeling. During tumor progression, N2 neutrophils could fuel both primary tumor growth and tumor metastasis [12]. Our data showed that IL-35 could induce the N2 phenotype of neutrophils by inducing the conversion of neutrophil function from tumor-suppressing to tumor-promoting. After the sustained expression of IL-35 *in vivo*, neutrophils produced more MMP-9 and Bv8 that promote tumor angiogenesis [38, 39] and iNOS that inhibits T cell activation, but expressed much less TRAIL that induces tumor cell apoptosis and inhibits angiogenesis [40, 41]. Therefore, in tumor-associated inflammation, IL-35 could be a crucial cytokine that controls the polarization of neutrophils.

IL-35 could not directly stimulate neutrophils to induce their functional conversion as shown by our data. Instead, the sustained IL-35 expression *in vivo* increased the production of G-CSF and IL-6. Our previous study showed that G-CSF and IL-6 can induce the conversion of neutrophil function from tumor-suppressing to tumor-promoting [13]. IL-6 cooperates with G-CSF to induce the sustained and enhanced activation of STAT3, thus up-regulating the expression of MMP-9 and Bv8, but down-regulating TRAIL expression in neutrophils [13]. In this study, we further demonstrated that G-CSF and IL-6 could upregulate the expression of iNOS that contributes to the survival and proliferation of tumor cells by suppressing T cell activation. ERK and STAT3 pathways were crucial for G-CSF to induce iNOS expression in neutrophils. IL-6 induced the expression of iNOS by activating STAT3. IL-6 cooperation with G-CSF could further increase the expression of iNOS. Moreover, our data in this study further confirmed that the enhanced STAT3 activation is crucial for tumor-promoting function of neutrophils. In line with the promoting effect of IL-35 on G-CSF/IL-6 production *in vivo*, the sustained expression of IL-35 could enhance the activation of STAT3 and ERK pathways in neutrophils. In this regard, the increased production of G-CSF/IL-6 is crucial for IL-35 to induce the N2 phenotype of neutrophils *in vivo*.

G-CSF and IL-6 could be produced by several types of cells *in vivo*. In this study, we demonstrated that macrophages were involved in IL-35-induced production of G-CSF and IL-6 *in vivo*. In response to IL-35 stimulation, IL-6 expression was directly induced in macrophages. Different from the expression of IL-6, the expression of G-CSF *in vivo* was induced by IL-35 through an indirect way. IL-17A has been known to induce the production of G-CSF *in vivo* [42]. Although IL-35 could suppress the differentiation of Th17 cells that produce IL-17A [3], our data showed that IL-35 could promote the expression of IL-17A *in vivo*. In this situation, IL-35 induced the expression of IL-17A in γδ T cells, since IL-35-induced expression of IL-17A could be suppressed by depleting γδ T cells. In the presence of tumor, γδ T cells, rather than Th17 cells, were predominant source of IL-17A [24]. Our data showed that IL-35 could induce the expression of IL-1β in macrophages, and that IL-1β further elicited the production of IL-17A from γδ T cells *in vivo*. Either blocking IL-1β or depleting γδ T cells could efficiently suppress IL-35-induced expression of G-CSF *in vivo*. Our data did not rule out the possibility that some other types of cells might be involved in IL-35-induced production of G-CSF/IL-6 *in vivo*. Nevertheless, our data clearly demonstrated that IL-35 could directly stimulate macrophages to produce IL-6, and induce G-CSF production through macrophages/IL-1β/γδ T cells/IL-17 axis.

High neutrophil numbers in the circulation in patients is associated with poor overall survival [43]. Our data showed that IL-35 could increase the proportion of neutrophils in peripheral blood. It is well known that G-CSF promotes myelopoiesis and mobilization of bone marrow neutrophils [42], which might be the main reason that IL-35 could increase the amount of neutrophils in blood circulation. In addition, IL-35 could also promote neutrophil infiltration in tumor microenvironment. As shown in our data, the genes of several chemoattractants for neutrophils were highly expressed in tumor, including *Cxcl2*, *Ccl2*, *Ccl3*, *Ccl4*, and *Ccl5*. In addition to *Ccl2*, the expression of these genes was not influenced by IL-35. Therefore, IL-35 might mainly promote the infiltration of neutrophils by increasing circulating neutrophils, based on the expression of multiple chemoattractants for neutrophils in tumor microenvironment.

During the development of tumor, IL-35 could be produced by not only tumor cells but also stroma cells and immune cells [44, 45]. Intriguingly, our data in this study showed that IL-35 could function as an up-stream cytokine to promote cancer-associated inflammation. The functional conversion of neutrophils is directly induced by G-CSF and IL-6. Importantly, the *in vivo* production of G-CSF and IL-6 could be augmented by IL-35. The sustained expression of IL-35 determines the conversion of neutrophil function from tumor-suppressing to tumor-promoting. Given that the protracted depletion of neutrophils is clinically untenable, targeting IL-35 might be very important for designing the therapeutic strategy to prevent the tumor-promoting effect of neutrophils.

## MATERIALS AND METHODS

### Animals and cell lines

Female BALB/c mice and C57BL/6 mice aged 6 to 8 weeks were purchased from the center of Medical Experimental Animals of Hubei Province (Wuhan, China). The mice were maintained in the accredited animal facility of Tongji Medical College, and animal care was in accordance with institutional guidelines. All animal experiments were approved by the Committee on the Ethics of Animal Experiments of Tongji Medical College (Permit Number: 2014-S514). The BALB/c background H22 hepatocarcinoma cell line and C57BL/6 background B16F0 melanoma cells were purchased from the China Center for Type Culture Collection (Wuhan, China) and cultured according to their guidelines.

### Reagents and plasmids

Murine rIL-35:Fc fusion protein was purchased from Chimerigen Laboratory (AdipoGen life science). The recombinant fusion protein possesses high stability and long circulating half-life owing to the extracellular domain of mouse IL-12a subunit is fused to the Fc region of human IgG1, and the mouse EBI3 subunit linked to IL-12a by disulfide bonds. Plasmid pIL-35 is the expression vector that expresses a “native” form of IL-35 made of IL-12a and Ebi3 linked subunits. pIL-35 (pUN01 IL-35 elasti) was purchased from Invivogen. Plasmid pUN01 was also purchased and used as control plasmid. Plasmids pG-CSF, pIL-6, and pCXCL1 are expression vectors carrying the cDNA encoding murine G-CSF, IL-6, and CXCL1, respectively. These plasmids were constructed by the insertion of cDNA into plasmid pcDNA3.1 (Invitrogen, Carlsbad, CA) in our laboratory [13].

### *In vivo* gene transfection

Plasmids were prepared and analyzed as described previously [46]. Mice received the injection of plasmid DNA (in 100 μl PBS) into the muscle tissue (i.m. injection) at the inoculation site or the injection of plasmid DNA via the tail vein (i.v. injection) using the hydrodynamics-based gene delivery technique [46].

### Animal experiments and treatment protocols

To acquire mice with *in vivo* expression of IL-35, mice received an i.m. injection of pIL-35 plasmids (100 μg of each per injection) in the left hind thigh (once every two days, four times); pUN01 plasmid was used as control. The mice were used for further experiment on d10 after first injection of plasmid. To acquire mice with *in vivo* expression of G-CSF and/or IL-6, mice received an i.m. injection of pG-CSF and/or pIL-6 plasmids (100 μg of each per injection) in the right hind thigh (once every two days, four times); pcDNA3.1 plasmid was used as control. Naive mice and the mice receiving plasmid injection were labeled by N, pUN01, pIL-35, pG, pI6, pG/pI6, and p3.1, respectively.

To recruit neutrophils to the inoculation site, mice were inoculated intramuscularly in the right hind thigh with 1×10^5^ H22 cells or 3×10^5^ B16 cells. The mice in treatment groups received an i.m. injection, once every 2d, of pCXCL1 (100 μg per injection, day 1 to day 5 after tumor cell inoculation). The p3.1 plasmid was used as control. Tumors were dissected and weighed on d10 after tumor cell inoculation.

In co-inoculation test, mice were inoculated intramuscularly in the right hind thigh with H22 or B16 cells, mixed with 1×10^6^ neutrophils. Tumors were dissected and weighed on d10 after tumor cell inoculation.

In intra-tumor injection test, the mice with palpable tumor were randomly divided into several groups. 1×10^6^ neutrophils or equal volume of PBS (50 μl) were carefully injected to tumor, once a day for three times. Tumors were dissected on d3 after last injection for analysis of microvessel density.

To analyze the neutrophils in tumor tissues, mice were inoculated intramuscularly in the right hind thigh with H22 cells or B16 cells. For detecting neutrophils by flow cytometry, single-cell suspensions of tumors were prepared. In brief, Tumors were harvested from mice, minced, and digested with 2 mg/ml DNase I (Sigma, St. Louis, MO) and 4 mg/ml collagenase type IV (Sigma) at 37°C for 1 h. After a PBS washing, cell suspensions were filtrated with a 200-mesh sieve for subsequent flow cytometry.

### Cytokine neutralization and γδ T cell depletion *in vivo*

For cytokine (IL-17A or IL-1β) neutralization experiments, mice were injected intraperitoneally with 50 μg twice weekly for anti-IL-17A (Clone 50104; R&D Systems), or 50 μg twice weekly anti-IL-1β (clone B122; BioXCell). To deplete γδ T cells *in vivo*, mice were injected intraperitoneally with an initial 400 μg followed by 100 μg twice weekly for anti-γδ TCR (clone GL3; R&D Systems). Control mice received equal amounts of isotype control antibodies or equal volumes of PBS.

### Recruitment of neutrophils to peritoneal cavity

To acquire CXCL1-expressing hepatocytes, mice received i.v. injection of pCXCL1 plasmid (200 μg per mouse). 12 h later, hepatocytes were prepared from liver by two-step collagenase perfusion technique [13]. To recruit neutrophils to peritoneal cavity, CXCL1-expressing hepatocytes were injected to peritoneal cavity of mice (3 × 10^5^ per mouse). 12 h later, the peritoneal cells were harvested for the isolation of neutrophils.

### Isolation of neutrophils

Murine neutrophils were isolated from bone marrow cells or peritoneal cells as described previously [13]. Briefly, the cells were washed once in HBSS media. Cells were separated by centrifugation over a 3 layer discontinuous Percoll gradient (54%/64%/72% for bone marrow cells and 54%/64%/80% for peritoneal cells). The dense band at 64%/72% or 64%/80% interface was collected as neutrophil fractions. Purity of isolated neutrophils was validated by flow cytometry and only samples greater than 90% purity was used (Supplementary Figure 6).

### Generation of murine bone marrow-derived macrophages

Murine bone marrow derived macrophages were obtained according to the protocol in previous publication [47]. Bone marrow cell suspensions were cultured in the DMEM medium supplemented with 10% fetal calf serum, 1% Penicillin/Streptomycin and 10 U/ml recombinant murine M-CSF (PepProTech). Macrophages were then harvested on day 7 for subsequent cell stimulation assays.

### Other methods

Other methods were performed using standard protocols, including cell transfection, flow cytometry and intracellular staining, assay of gene expression by real-time RT-PCR, immunohistochemistry, MMP-9 assay, Western blot assay, ELISA analysis, T cell proliferation assay. See Supplemental Methods for details.

### Statistical analysis

Results were expressed as mean value ± SD and interpreted by one-way ANOVA. Differences were considered statistically significant when *P* < 0.05.

## SUPPLEMENTARY MATERIALS FIGURES


