# Supplementary material for: IL-35 induces N2 phenotype of neutrophils to promote tumor growth
Source: Oncotarget. 2017 Apr 4;8(20):33501–14. doi: 10.18632/oncotarget.16819 (PMC5464885; doi:10.18632/oncotarget.16819)
Supplement: Supplementary file 1 [file oncotarget-08-33501-s001.pdf]

IL-35 induces N2 phenotype of neutrophils to promote tumor growth

Supplementary Materials

Cell transfection

To express IL-35 in B16F0 cells, the cells were transfected with plasmid pIL-35 with Lipofectamine® 2000 transfection reagent (Invitrogen, Carlsbad, CA). As a control, B16F0 cells were transfected with plasmid pUN01. The stable cell lines resistant to blasticidin were generated, and designated as B16-IL35 and B16-Ctrl respectively. The expression of IL-35 in B16-IL35 cells was identified by RT-PCR and ELISA (Supplementary Figure 7). The sequences of the primers for RT-PCR were as follows: recombinant IL-35, sense 5′-TTCTCCACGGTGCCCTACAT-3′, anti sense 5′-TGAAGGCGTGAAGCAGGATG-3′; EBI3, sense 5′-TTCTCCACGGTGCCCTACAT-3′, antisense 5′-AGGC TCCAGTCACTTGG TTTCC-3′; IL-12a, sense 5′-AC GGGACCAAACCAGCACAT-3′, antisense 5′-TGAAGG CGTGAAGCAGGATG-3′; β-actin, sense 5′-AGGGAAA TCGTGCGTGAC-3′, antisense 5′-C GCTCGTTGCC AATAGTG-3′.

Flow cytometry and intracellular staining

The following antibodies were used for cell surface and intracellular staining; PE-Cy7-labeled anti-CD11b, PerCP-Cy5.5-labeled anti-IL-17A, FITC-labeled anti-CD4, PE-labeled anti-Ly6G from BD Biosciences. For intracellular staining, cells were incubated with permeabilization buffer (BD Biosciences), and then further stained with intracellular Abs described above. These cells were analyzed by using LSRII flow cytometer (BD Bioscience) and FlowJo software.

Assay of gene expression by real-time RT-PCR

Total RNA was isolated from cells by TRIzol extraction (Invitrogen). The relative quantity of mRNA was determined by real-time RT-PCR. β-actin was chosen as reference genes. The relative expression of gene was calculated using GeNorm software. The primer sequences were as follows: CXCL1, sense 5′-ACCCAAACCGAAGTCATAGC-3′, antisense 5′-AG AAGCCAGCGTTCACCAGA-3′; CXCL2, sense 5′-CCC AGACAGAAGTCATAGC -3′, antisense 5′-TCCTTT CCAGGTCAGTTAG-3′; CXCL5, sense 5′-CTGGCAT TTCTG TTGCTGTT-3′, antisense 5′-TATGACTTCC ACCGTAGGGC-3′; CXCL15, sense 5′-TA TTCCCGC GTTAGTCTGGTG-3′, antisense 5′-GCCCATAGTGGA GTGGGATAAG-3′; CCL2, sense 5′-TGGGTCCAGA CATACATT-3′, antisense 5′-ACGGGTCAACTTCAC AT T-3′; CCL3, sense 5′-ATTCCACGCCAATTCATC-3′, antisense 5′-GCATTCAGTTC CAGGTCA-3′; CCL4, sense 5′-TTGCTCGTGGCTGCCTTCT-3′, antisense 5′-AC TGCTG GTCTCATAGTAATC-3′; CCL5, sense 5′-AC CACTCCCTGCTGCTTT-3′, antisense 5′-AC ACTTGG CGGTTCCTTC-3′; VEGF, sense 5′-ATGGATGTCTA CCAGCGAA-3′, antisense 5′-CACGCACTCCAGGG CTTC-3′; MMP-9, sense 5′-TGCCCAGCGACCACAA CTC-3′, antisense 5′-CGGACCCGAAGCGGACATT-3′; Bv8, sense 5′-TGCTACTTCTGCTGCTAC C-3′, anti sense 5′-CCGCACTGAGAGTCCTTGTC-3′; TRAIL, sense 5′-TACTGGGATCAC TCGGAGAAG-3′, anti sense 5′-ACGTGGTTGAGAAATGAATGCC-3′; G-CSF, sense 5′-A GGGAAGGAGATGGGTAAAT-3′, antisense 5′-CGGAAGGGAGACCAGATGC-3′; IL-6, sense 5′-GCT GGAGTCACAGAAGGAG-3′, antisense 5′-TAGGTTT GCCGAGTAGA-3′; Arg-1, sense 5′-TGGGAAGA CAGCAGAGGA-3′, antisense 5′-TCAGTCCCTGGCT TAT GG-3′; Nos2, sense 5′-GGAGCGAGTTGT GGATTGTC-3′, antisense 5′-TGAGGGCTTGG CTGAG TGA-3′; IL-17A, sense 5′-TGTCCAAACACTGAG GC-3′, antisense 5′-AACGGT TGAGGTAGTCTGA-3′; IL-1β, sense 5′-TTCCCATTAGACAACTGC-3′, antisense 5′-GGT ATAGATTCTTTCCTTT-3′; TGF-β1, sense 5′-GGACCCA ACAACGCCATCT-3′, antisense 5′-AA GCGCAGCTCTGCACGGGACA-3′.

Immunohistochemistry

Tissue sections were prepared and subjected to immunohistochemical analysis as described previously [46]. Anti-mouse CD34 Ab (Santa Cruz Biotechnology) was used as primary Abs for detecting microvessel. HRP-conjugated secondary Ab was used for detecting the microvessel in the H22 tumor. Cy3-conjugated

secondary Ab was used for detecting microvessel in the B16 melanoma. Images were obtained using an Olympus-IX71 microscope at 40 × 10 magnification. Criteria for positive-staining microvessel counting followed the method described by Weidner [48]. Microvessel density was defined as the number of microvessel per 0.2 mm^2.

MMP-9 assay

To detect neutrophil-released MMP-9, neutrophils were incubated at the concentration of 5×10^6/ml at 37 °C for 4 h in RPMI 1640 medium. MMP-9 in supernatants was detected by gelatin zymography, and the relative activity of MMP-9 was calculated as described previously [49].

Western blot assay

The indicated cells were isolated from mice. Western blot assay was done as described previously [50]. Primary antibodies and horseradish peroxidase-conjugated secondary antibodies were purchased from Santa Cruz Biotechnology (Santa Cruz, CA) and Cell Signaling (Beverly, MA).

ELISA analysis

A mouse IL-35 ELISA kit (LEGEND MAXTM, BioLegend, San Diego, CA) was used to detect IL-35 in the culture supernatants of B16-IL35 cells.

The levels of IL-17A, G-CSF, IL-6 or IL-1β in serum or tumor microenvironment were detected using mouse IL-17A, G-CSF, IL-6 or IL-1β ELISA kits (R&D systems, Minneapolis, MN) at the indicated time point.

Macrophages were incubated in advanced RPMI 1640 medium (Invitrogen) supplemented with L-glutamine (Gibco, Carlsbad, CA, USA), and stimulated for 12 h with the following agents: IL-35 (mouse):Fc (human) recombinant protein (50, 100, 200 ng/ml). IL-6, G-CSF and IL-1β released into the cell culture supernatants were measured by ELISA kits. The absorbance was measured at 450 nm using an ELISA reader (Tecan Sunrise, Salzburg, Austria).

To detect G-CSF, IL-6, and IL-17A in tumor, B16-IL35 cells were intramuscularly inoculated to C57BL/6 mice. Alternatively, H22 cells were intramuscularly inoculated to BALB/c mice. The mice then received the injection of plasmid pIL-35 DNA (in 100 µl PBS) into the muscle tissue (i.m. injection) at the inoculation site, once every two days starting from d1 after tumor cell inoculation. On d14 after tumor cell inoculation, tumors were dissected and homogenized. G-CSF, IL-6, and IL-17A were detected by ELISA. The results were expressed as pg cytokine/mg tissue protein.

T cell proliferation assay

Bone marrow neutrophils were isolated from mice. Splenic cells from naive mice were isolated and labelled with Cell Trace CFSE following the manufacturer's instructions (Invitrogen). Equal numbers of cells (4×10^5) were co-cultured in a 96-well flat bottom plate. CD3/CD28 Dynabeads (Invitrogen) were added according to manufacturer's instruction. L-NMMA (iNOS inhibitor, Sigma) and norNOHA (arginase1 inhibitor, Sigma) were added at 0.5 mM where indicated. After 48-h culture, splenic cell proliferation was evaluated on a BD LSRII flow cytometer using Diva software. Data were analyzed by using FlowJo Software version 9.7.1. Proliferation index was calculated using the formula: (percentage of proliferated, co-cultured splenic cells)/(percentage of proliferated splenic cells without co-culture) ×100, for each replicate experiment.

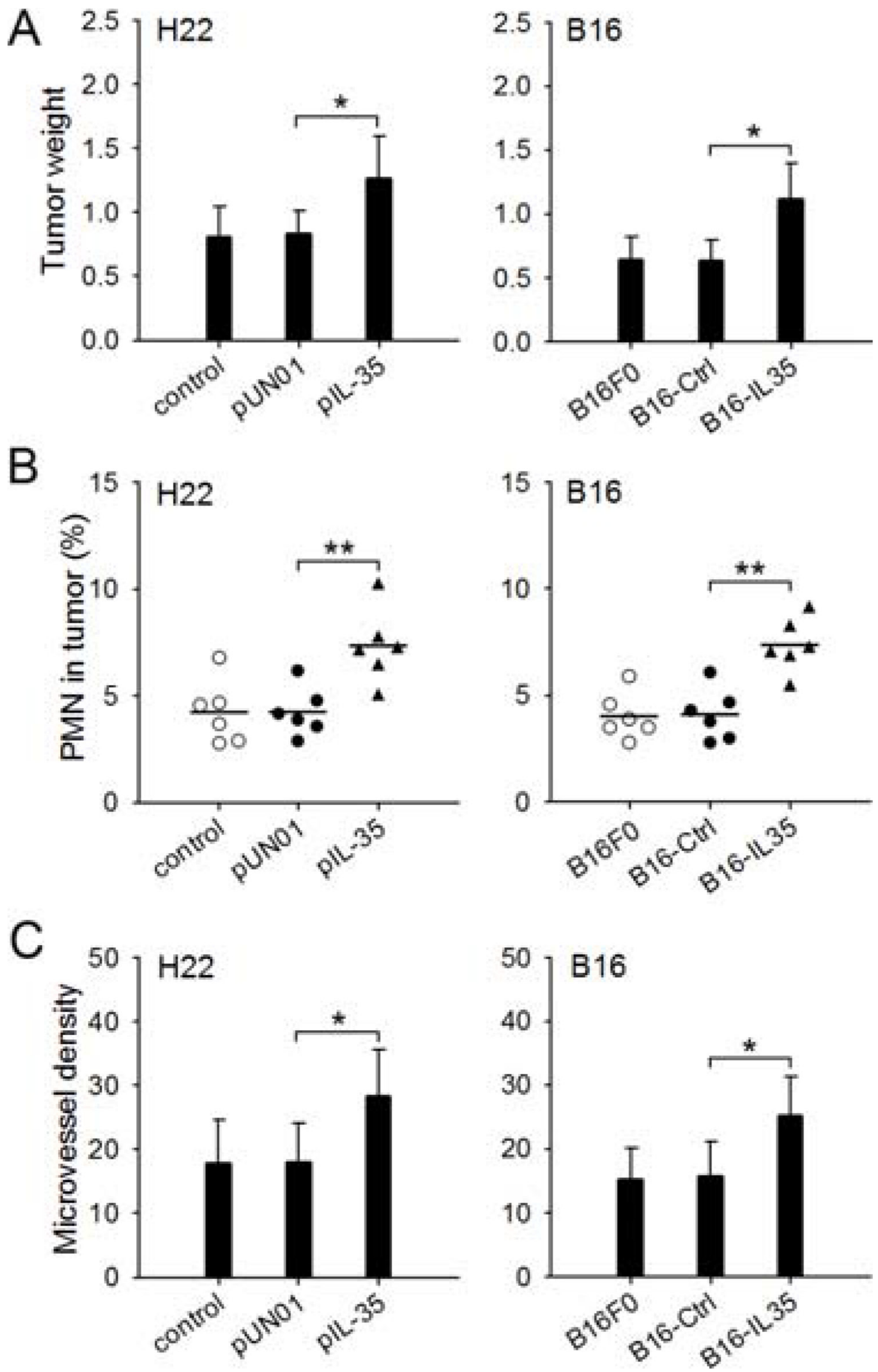

Supplementary Figure 1: Local IL-35 expression promotes neutrophil infiltration and tumor progression. B16F0, B16-Ctrl, and B16-IL35 cells were intramuscularly inoculated to the right hind thigh of C57BL/6 mice. H22 cells were intramuscularly inoculated to the right hind thigh of BALB/c mice, followed by the intramuscular injection of pIL-35 at the inoculation site, once every two days starting from d1 after tumor cell inoculation. Tumors (n = 6 per group) were dissected on d14 after inoculation. (**A**) Tumors were weighed. (**B**) Neutrophils in tumor tissues were analyzed by flow cytometry. (**C**) On d7 after tumor cell inoculation, CXCL1 was expressed in local tissues to recruit neutrophils, as described in Methods. Tumors were dissected on d14 after inoculation. Microvessels in tumor tissues were detected by immunohistochemical analysis. Microvessel density was determined as described in Methods. *$p < 0.05$, **$p < 0.01$.

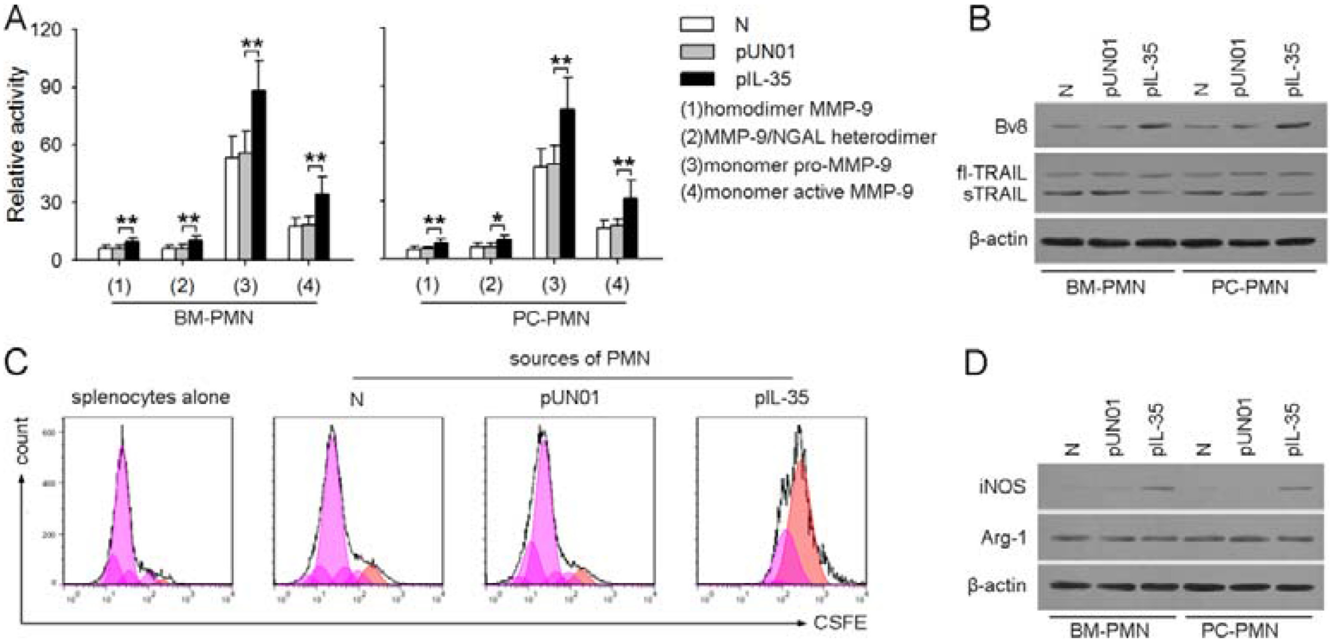

Supplementary Figure 2: IL-35 augments proangiogenic and immunosuppressive function of neutrophils *in vivo*. Neutrophils were isolated from bone marrow (BM) or peritoneal cavity (PC) of N-mice, pUN01-mice, and pIL-35-mice. (**A**) The release of MMP-9 from neutrophils was detected by zymography as described in Methods. (**B**) The expressions of Bv8, full-length (fl) and soluble (s) TRAIL were detected by Western blot. (**C**) CFSE-labeled splenocytes from naive mice were incubated with CD3/CD28 stimulation beads in the absence or presence of the neutrophils isolated from bone marrow of N-mice, pUN01-mice and pIL-35-mice. After 48-h culture, CFSE dilution was evaluated by flow cytometry. Representative histograms of splenocyte proliferation were showed from three independent experiments with similar results. (**D**) Neutrophils were isolated from bone marrow or peritoneal cavity of N-mice, pUN01-mice, and pIL-35-mice.The expression of iNOS and Arg-1 in neutrophils was detected by Western blot. Data are pooled from three independent experiments with a total six samples in each group (A), or are representative of three independent experiments (B, C, D). *$p < 0.05$, **$p < 0.01$.

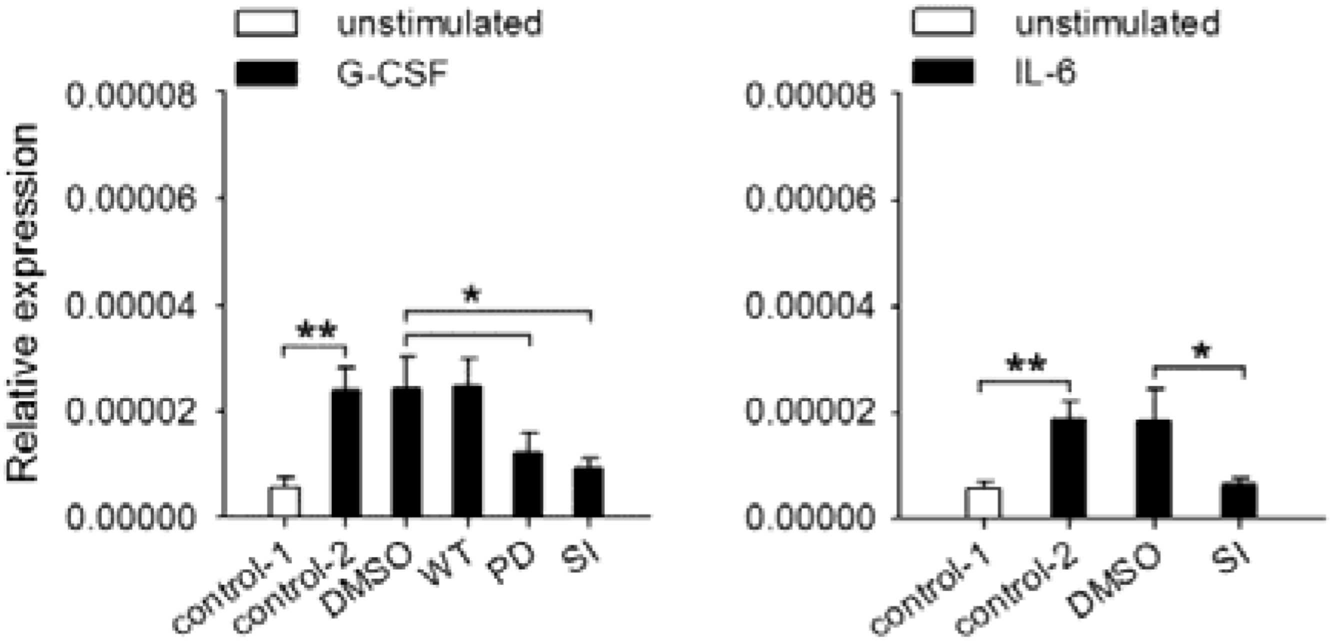

Supplementary Figure 3: Enhanced activation of STAT3 and ERK pathways in neutrophils is required for the upregulation of Nos2 gene. Neutrophils were isolated from bone marrow of naive mice. The cells (2×10^6/ml) were untreated or pretreated for 2 h with STAT3 inhibitor VIII (SI, 50 μM), wortmannin (WT, 10 nM), PD98059 (PD, 20 μM). Then, the cells were unstimulated or stimulated for 12 h with G-CSF or IL-6 (50 ng/ml of each) in the absence or presence of the same inhibitor. The expression of *Nos2* gene was detected at the mRNA level by real-time RT-PCR. Data are pooled from three independent experiments with a total six samples in each group. *$p < 0.05$, **$p < 0.01$.

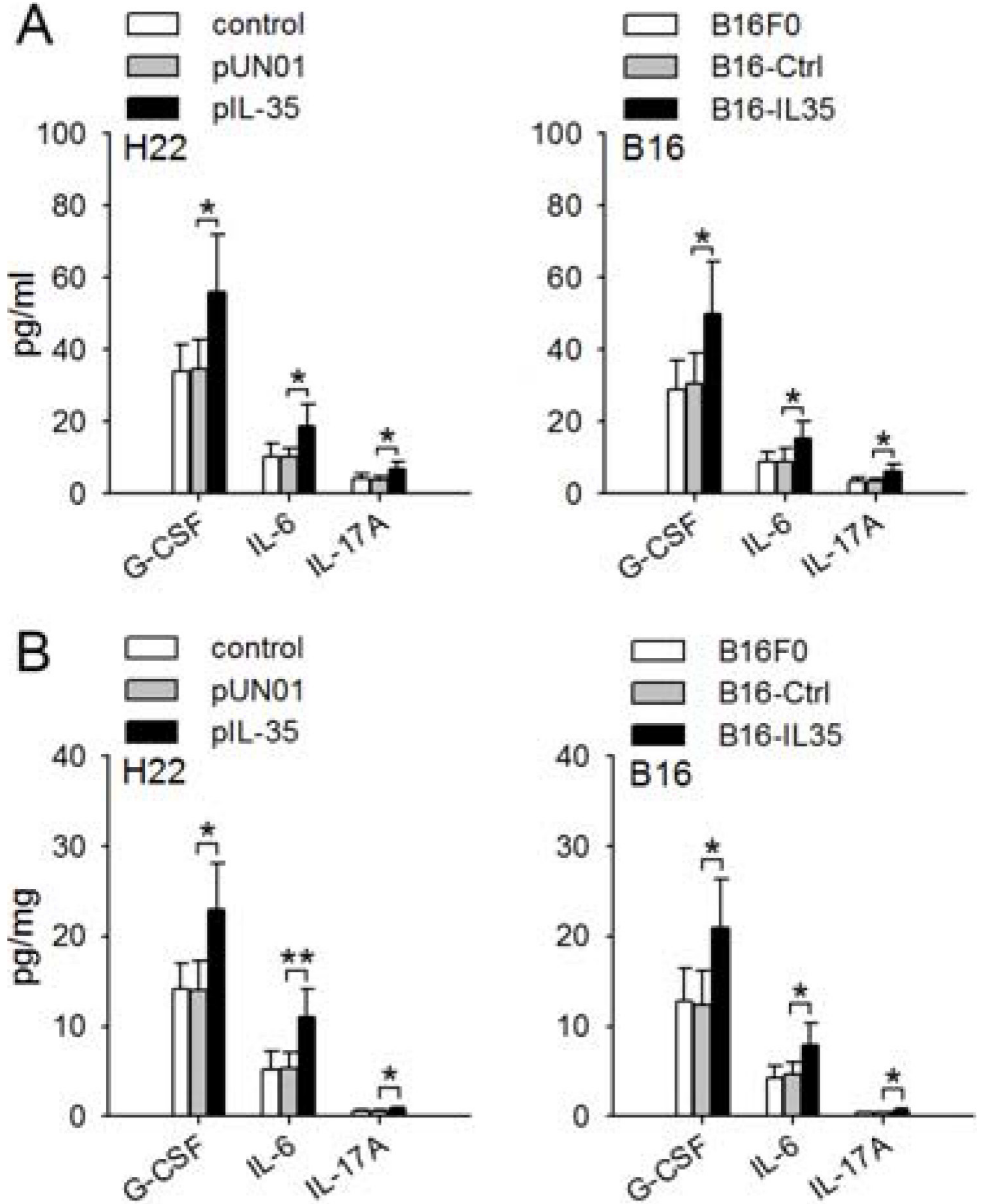

Supplementary Figure 4: Local IL-35 expression increases G-CSF, IL-6 and IL-17A production in blood and tumor microenvironment. B16F0, B16-Ctrl, and B16-IL35 cells were intramuscularly inoculated to the right hind thigh of C57BL/6 mice. H22 cells were intramuscularly inoculated to the right hind thigh of BALB/c mice, followed by the intramuscular injection of pIL35 at the inoculation site, once every two days starting from d1 after tumor cell inoculation. Tumors (n = 6 per group) were dissected on d14 after tumor cell inoculation. Sera were collected at the same time. (**A**) ELISA analysis of G-CSF, IL-6 and IL-17A in the serum. (**B**) ELISA analysis of G-CSF, IL-6 and IL-17A in the tumor tissues. *$p < 0.05$, **$p < 0.01$.

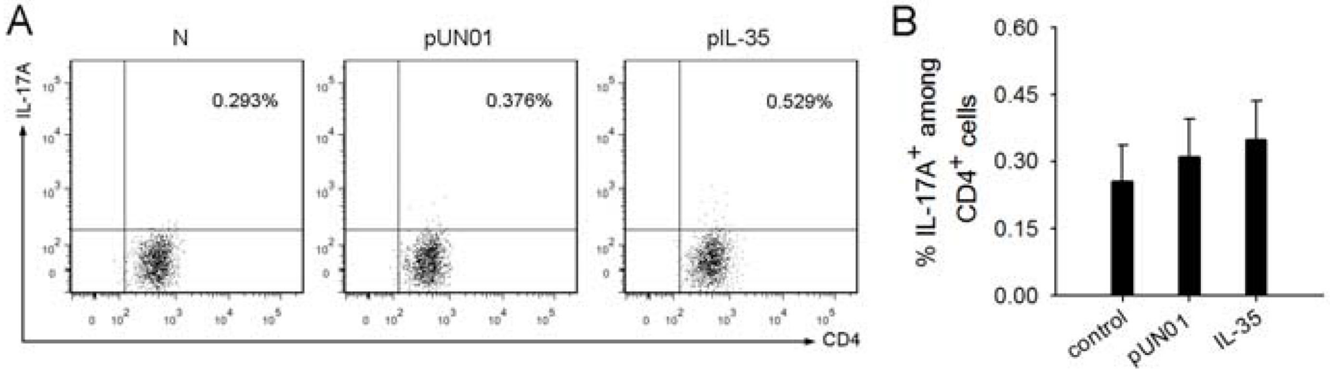

Supplementary Figure 5: Effect of IL-35 treatment on differentiation of Th17 cells in spleen. (**A**) Representative dot-plot showing IL17^{+} on gated CD4^{+} cells in naive mice, pUN01-mice and pIL-35-mice. (**B**) Frequency of Th17 cells among CD4^{+} cells. Data are representative of three independent experiments (A), or pooled from three independent experiments with a total six samples in each group (B).

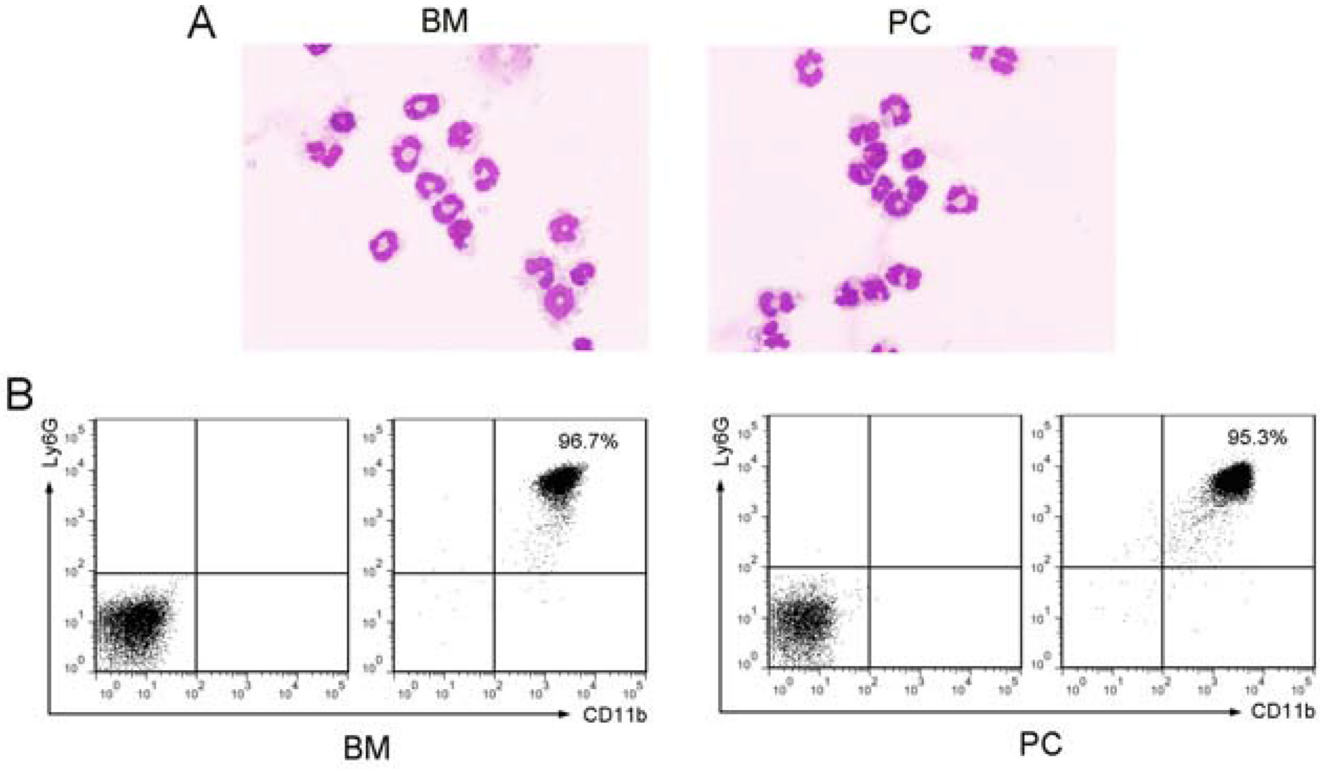

Supplementary Figure 6: Analysis of the isolated neutrophils. (**A**) Neutrophils were isolated from bone marrow (BM) and peritoneal cavity (PC) of mice. The cells were identified by Giemsa-Wright stain. (**B**) The cells were stained with PE-Cy7-anti-mouse CD11b and PE-anti-mouse Ly6G antibodies, and used for flow cytometry.

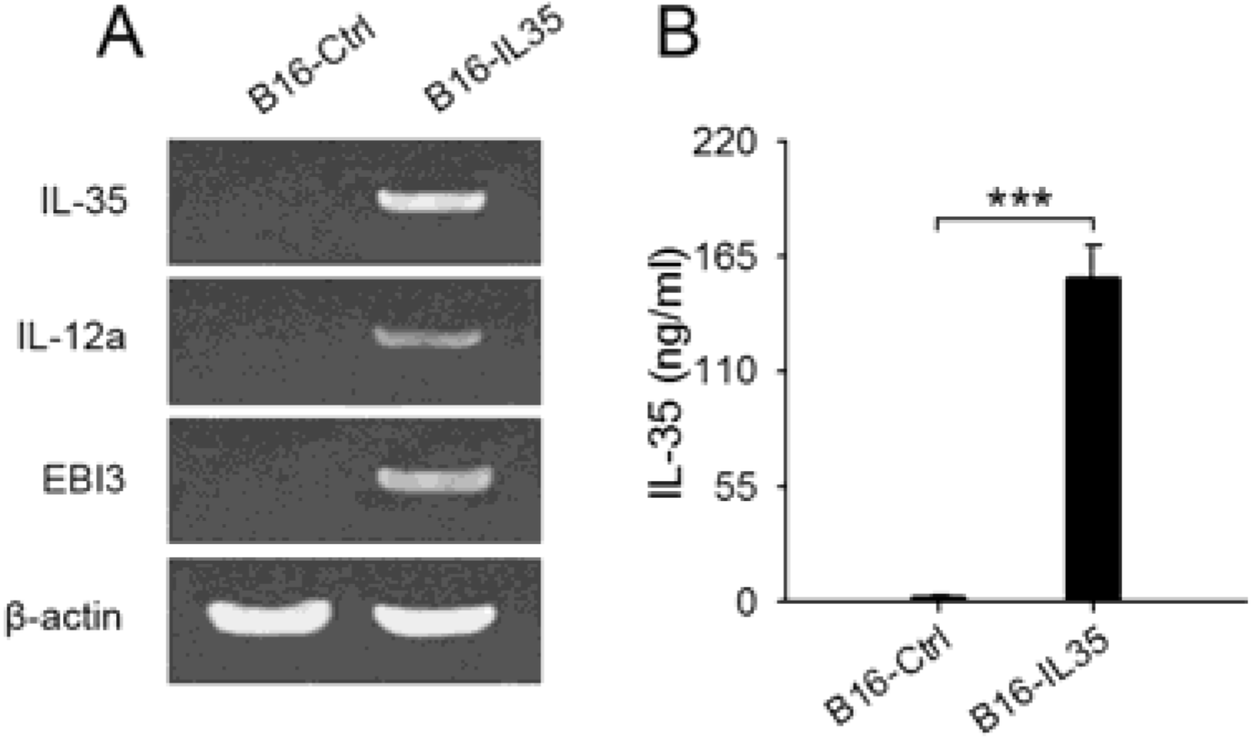

Supplementary Figure 7: Expression of IL-35 in B16 cells after transfection of expression vector. B16F0 cells were transfected with an expression vector of IL-35 (pUN01 mIL-35 elasti) or the control vector pUN01. Stable cell lines resistant to blasticidin were generated. (**A**) RT-PCR was used to detect the expression of transcripts for recombinant IL-35, IL-12a and EBI3. (**B**) ELISA was used to detect IL-35 in the culture supernatants of the cells. ***$p < 0.001$.
